# A multi-pattern hash-binary hybrid algorithm for URL matching in the HTTP protocol

**DOI:** 10.1371/journal.pone.0175500

**Published:** 2017-04-11

**Authors:** Ping Zeng, Qingping Tan, Xiankai Meng, Zeming Shao, Qinzheng Xie, Ying Yan, Wei Cao, Jianjun Xu

**Affiliations:** 1 College of Computer, National University of Defense Technology, Changsha, Hunan, P.R. China; 2 National Key Laboratory for Parallel and Distributed Processing, National University of Defense Technology, Changsha, Hunan, P.R. China; 3 School of Computer and Communication Engineering, Changsha University of Science and Technology, Changsha, Hunan, P.R. China; Universita degli Studi di Catania, ITALY

## Abstract

In this paper, based on our previous multi-pattern uniform resource locator (URL) binary-matching algorithm called HEM, we propose an improved multi-pattern matching algorithm called MH that is based on hash tables and binary tables. The MH algorithm can be applied to the fields of network security, data analysis, load balancing, cloud robotic communications, and so on—all of which require string matching from a fixed starting position. Our approach effectively solves the performance problems of the classical multi-pattern matching algorithms. This paper explores ways to improve string matching performance under the HTTP protocol by using a hash method combined with a binary method that transforms the symbol-space matching problem into a digital-space numerical-size comparison and hashing problem. The MH approach has a fast matching speed, requires little memory, performs better than both the classical algorithms and HEM for matching fields in an HTTP stream, and it has great promise for use in real-world applications.

## Introduction

Multiple-pattern string matching algorithms based on uniform resource locator (URL) rule sets are widely used in firewall, network traffic analysis, data acquisition, web server load balancing, firewall blacklists, e-mail classification, spam detection, intrusion detection, URL-based content classification [1], and other fields. In recent years, the rapid development of cloud computing, big data, and artificial intelligence has also greatly promoted developments in robotics. Robotics research has widened from an original focus on a single robot to areas of controlling multiple robots simultaneously, into swarm robotics [2], network robotics [3] and cloud robotics [4–7]. Just like the web, these robotics areas have similar requirements for performing URL or string matching based on specific protocols. As the network data-flow rate has increased year over year, each of these areas require an algorithm that can conform to tens of thousands or even millions of rules while still achieving a processing capacity of 10 Gbps. Unfortunately, the classical multi-pattern string matching algorithms are unable to keep up with such demands. Therefore, we conducted a series of explorations into multi-pattern string matching based on the characteristics of the HTTP protocol.

### Introduction to the HTTP protocol

The hypertext transfer protocol (HTTP), which is based on the TCP protocol, was first proposed in 1990. HTTP is an application layer protocol. In 1999, the HTTP 1.1 version was launched and has since undergone further development and improvement. The simple and convenient characteristics of the HTTP protocol made it superior for expressing a variety of media resources and transmission types; therefore it has been the basis for a great deal of development. This article uses only the pertinent parts of the protocol.

In HTTP 1.1, messages are divided into two types—request and response messages; however, our algorithm is concerned mainly with request messages in practical applications. In these request messages, the methods, resource indicators, protocol version, and so on are contained in the first line. Two fields, named Request-URI and Host header in the request messages, identify the request resources; the Host header field can be ignored if the Request-URI is an absolute address [8].

In most cases, our algorithm focuses primarily on the non-absolute address Request-URI in which the Host header need not be considered. To apply our algorithm to absolute address Request-URI requests, we can obtain the address using a low time complexity simple traversal and split it into a string that has the same format as a non-absolute address Request-URI. The HTTP 1.0 version usually does not contain the Host header field; consequently, the Host header field can be considered as a null value and combined with the destination IP address and port in a 5-tuple for processing.

### Related work

Multi-pattern string matching refers to the process of determining the applicable rule subsets for target strings or data streams from a rule set that contains multiple string rules. At present, the main multi-pattern string matching algorithms include prefix, suffix and substring matching algorithms. For instance, the AC [9] algorithm and the SBOM [10] algorithm are based on automata, the WM [11] algorithm is based on a hash, the M-BNDM [12] algorithm is an extension of the BNDM [13] algorithm, which is based on bit parallels, and so on.

However, every classical algorithm has its limitations: the automata-based algorithms often have large storage requirements, which is unacceptable in embedded systems or in wearable devices or robots that have limited hardware resources. Moreover, such algorithms often support only small-scale string pattern rules. Classical hash algorithms have fast matching speeds, but lead to conflicts in URL pattern sets that follow particular distributions, causing performance degradation. Although reducing the load factor can reduce conflict, doing so also wastes storage. The bit-parallel algorithms are restricted to the target machine's word width, and their adaptability to rule scope is limited, often supporting only dozens of string rules. Single pattern matching algorithms (such as the BM [14] algorithm) have excellent performance; however, they are unable to meet the performance demands for matching multi-pattern fields.

Due to the limitations of the classical algorithms, researchers have explored some newer multi-pattern matching algorithms. For example, He et al. [15] proposed a string matching algorithm with an optimal time complexity; Hlayel and Hnaif [16] proposed a DMA algorithm to improve matching performance; Faro et al. [17] proposed a fast short-form string matching algorithm, EPSM, based on the Intel SIMD Flow Instruction Extension (SSE) technique; Al-Ssulami [18] proposed an SSM algorithm based on Horspool; and Aldwairi et al. [19] used the Bloom filter to improve the WM algorithm, and presented the EXHAUST algorithm, which reduced the number of searches for large hash tables. In addition, Faro et al. [20] developed the string matching algorithm research framework called Smart, which implements BXS [21], BP2WW [22], KBNDM [23], SSECP [24], FSBNDM [25], BSDM [26] and other string matching algorithms. These works have a good effect on general text or corpus matching, but in direct application to URL-matching in the field, their performance is obviously insufficient.

To meet the bottleneck of traditional general string algorithm matching performance, much exploration and research has been conducted in the URL matching field: Li and Feng [27] presented two effective functions from a hash perspective; Liu et al. [28] proposed the SOGOPT algorithm, which is based on the SOG algorithm; He et al. [29] explored applying hash-based URL classifications to distributed search engines; and Bremler-Barr et al. [30] proposed a low-storage and low-time-overhead URL-matching framework called SUMSMF which was based on dictionary compression. However, these works still adopt the traditional string matching approach to improve URL matching, and are not well suited to the characteristics of the HTTP protocol or to the digital characteristics of the strings; therefore, their ability to improve performance in this area is still limited.

Based on specific application requirements, the characteristics of URL strings are effectively combined with the HTTP protocol and the machine word width in the proposed efficient matching algorithm called HEM [31], which is based on multilayer binary tables. Experiments shows that the HEM algorithm can overcome the deficiencies of the classical algorithms and it exhibits high efficiency, easy configuration, space stability, and so on, is well suited to HTTP data-flow matching and field filtering. However, the time complexity of this algorithm tends to *O(mlogn)*, which is still not optimal. Therefore, a modified hash-binary hybrid URL-matching algorithm called the MH algorithm is further proposed.

### Text structure

The MH algorithm is introduced in detail in Section 1, which also contains a discussion of the patterns involved in both the preprocessing and matching processes. The algorithm's time and space complexity are also analyzed from a theoretical point of view. Section 2 presents several experiments that demonstrate the superiority of the MH algorithm. Finally, Section 3 summarizes and analyzes our algorithm's performance and discusses plans for future research.

### Term definitions

This paper includes the following terms and definitions:

**HOST:** The HOST identifier in the URL. In this paper, the HOST string does not include the trailing "/" character.

**PATH:** The portion of the URL after the first "/" character. For pure HOSTs without the trailing "/" character, the PATH is empty; however, in this study, for convenience, we set the PATH to "/" when the PATH was empty.

**Integer(expression):** A function that rounds the expression up to the next highest integer value. The result of this operation on the expression in parentheses is an integer; the expression itself can be any pure number. For example, the result of Integer(1.1) is 2.

**End node:** The corresponding PATH or HOST node of the last PATH part or last HOST part in a rule. The end node may not necessarily be the last PATH node or HOST node. For example, if the PATH part of rule R1 is the common prefix substring of rule R2, the last node of the PATH part of rule R1 is an End node but is not the final PATH node (because R2 uses the subsequent PATH nodes).

**Pattern mutex:** For a pattern set *Pt = {p*^*1*^, *p*^*2*^, *……*, *p*^*n*^*}*, if any of the pattern strings *p*^*i*^*(1 ≤ i ≤ n)* appear in the target data stream *T* and none of the other pattern strings *p*^*j*^*(1 ≤ j ≤ n*, *and j ≠ i)* appear in *T*, then the pattern string in the pattern set *Pt* can be defined as a pattern mutex, and the pattern set *Pt* can be defined as a mutex set.

**Common prefix node:** For two rules with common nodes, rule *R1 = {p*^*1*^, *p*^*2*^, *h*^*1*^*}* and rule *R2 = {p*^*1*^, *p*^*2*^, *h*^*1*^, *h*^*2*^*} (h*^*1*^ *≠ h*^*2*^*)*, the two common nodes *p*^*1*^ and *p*^*2*^ are defined as common prefix nodes.

**Common end node mismatch problem:** For two rules with common nodes, rule *R1 = {p*^*1*^, *p*^*2*^, *h*^*1*^*}* and rule *R2 = {p*^*1*^, *p*^*2*^, *h*^*1*^, *h*^*2*^*}*, the three common nodes *p*^*1*^, *p*^*2*^, *and h*^*1*^ are defined as common end nodes. When the target string data stream *S = {p*^*1*^, *p*^*2*^, *h*^*1*^, *h*^*3*^*} (h*^*3*^ *≠ h*^*2*^*)* arrives, matching up to node *h*^*1*^ matches rule *R1*, but also matches rule *R2*. Therefore, as we continue to match the follow-up data, there will be a mismatch for node *h*^*2*^. Consequently, the algorithm must backtrack to *h*^*1*^. This problem is termed the common end node mismatch problem.

## Algorithm details

The MH algorithm is divided into three parts: the first part involves pattern preprocessing to construct hash-binary table chains segmented by machine word; the second part involves locating the PATH field and HOST field for the target HTTP1.1 data stream; and the third part is the fast matching process using the hash and binary search methods.

### Pattern preprocessing: Construct hash-binary table chains

In the HTTP protocol 1.1 version, the PATH and HOST fields are separate, and the PATH field occurs before the HOST field. In addition, a machine-word-length (*m*) memory space can store up to *m/8* ASCII characters. Based on this, we can divide the URL can be divided into the PATH string and HOST string following the method in [31] and then those are digitized.

#### Pattern segmentation and digital processing

We divide the URL into PATH and HOST by finding the first "/" character. The URL rules set *Pu = {u*^*1*^, *u*^*2*^, *……*, *u*^*n*^*}* containing *n* URLs can be divided into a PATH patterns set *Pp = {p*^*1*^, *p*^*2*^, *……*, *p*^*n*^*}* and a HOST patterns set *Ph = {h*^*1*^, *h*^*2*^, *……*, *h*^*n*^*}*. Then, we can divide the *Pp* and *Ph* sets based on the machine word length. In other words, each subpattern *p*^*i*^ of length *l(i)* in *Pp* can be divided into $\sum_{i=1}^{n}\frac{l(i)}{m}=\sum_{i=1}^{n}sp(i)$ machine words, and each subpattern *h*^*i*^ of length *l(i)* in *Ph* can be divided into $\sum_{i=1}^{n}\frac{l(i)}{m}=\sum_{i=1}^{n}sh(i)$ machine words. Finally, we obtain the basic rule sets *Pd = {{Pp*^*’*^, *Ph*^*’*^*}} = {{Cp11*, *Cp12*, *……*, *Cp1sp(1)*, *Ch11*, *Ch12*, *……*, *Ch1sh(1)}*, *{Cp21*, *Cp22*, *……*, *Cp2sp(2)*, *Ch21*, *Ch22*, *……*, *Ch2sh(2)}*, *……*, *{Cpn1*, *Cpn2*, *……*, *Cpnsp(n)*, *Chn1*, *Chn2*, *……*, *Chnsh(n)}}*. The number of sub-patterns in *Pp* or *Ph* is *sp(x) = Integer(l(x) / m)* where the length is *l(x)*.

These characters stored in machine words can be regarded as an *m*-bit-wide integer *I(0 ≤ I ≤ 2*^*m*^*-1)*. By regarding them in this manner, the string storage is transformed into integer storage, and the characters in the original sets *Pp* and *Ph* have been transformed into $sd(i)=\sum_{i=1}^{n}\left(sp(i)+sh(i)\right)$ machine words in the set *Pd*. Thus, the string matching problem in symbol space has been transformed into a numerical comparison problem in digital space.

#### Hash-binary table chains

**Fig 1:**
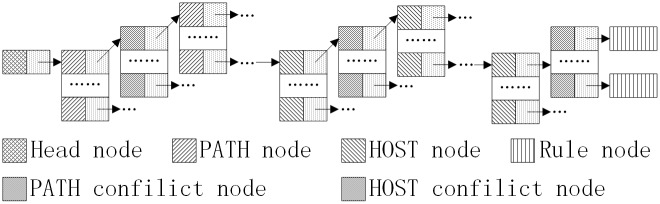
Complete structure of the MH hash-binary table chains.

The hash-binary table chains we established are special data structures formed by links in accordance with the head node, the hash table of the PATH, the conflict-binary table of the PATH, the hash table of the HOST, the conflict-binary table of the HOST and a sequence of rule nodes. The complete data structure is shown in Fig 1.

The hash-binary table chains have only one head node that contains two domains, one points to the first PATH hash table and the other is used to record the keyword of the first PATH hash table. Each node in the PATH hash table point to a subsequent PATH conflict-binary table with the same keyword. The nodes in the conflict-binary table point to the next PATH hash table in a chain that continues until the end of the PATH field; the nodes of the last PATH table for each rule point to a corresponding HOST hash table, which points to a subsequent HOST conflict-binary table and then to the next HOST hash table in a chain that continues until the end of the HOST field. Finally, the nodes of the last HOST table point to a corresponding rule node that stores the Rule ID.

In this data structure, each hash table contains a specific keyword that is stored in the corresponding node in the front layer. If a node has a conflict in the hash table, then the node will point to a conflict table that stores the same keyword data in which the data are stored in ascending order. In this way, a large-scale binary table can be decomposed into many smaller binary tables, which reduces matching times.

Due to conflicts and the common end node mismatch problem between rules, each PATH node must have nine domains as follows: (1) stores the PATH node (a machine word-length pattern string); (2) stores the PATH hash table address in the next layer; (3) stores the minimum mask of the PATH hash table in the next layer; (4) stores the corresponding HOST hash table address; (5) stores the minimum mask of the HOST hash table; (6) stores the number of nodes that have the same keyword in the conflict-binary table; (7) stores the conflict-binary table address; and (8) and (9) store the keyword of the PATH hash table and HOST hash table in the next layer, respectively. HOST nodes require only seven domains that store the address, keyword, and the minimum mask of the HOST hash table in the next layer, the HOST node information, the number of nodes in the conflict-binary table, the address of the conflict-binary table, and the rule node.

For PATH nodes, when the number of nodes in the PATH table and the HOST table in the next layer is greater than 0, the node is a common end node. For the HOST nodes, if the number of nodes of the HOST table is greater than 0 and the rule node address is not null, then the node is also a common end node. Note that common end nodes may be subject to common end node mismatch problems.

#### Simple hash method

We need to perform a hash operation on the target data when creating or searching the hash table. The MH algorithm uses a simple hash method: We set the initial keyword to *N* (*N* is the data number in the current hash table) when adding data, then perform modulo *N* on the target data and record the maximal count of the conflict data. When no conflict occurs, we set the final keyword to *N* and save the data into the table; otherwise, we set the keyword to *N + 1* and repeat the above operation until we find a keyword with no conflict. This method can reduce conflicts at small data scales, but for large-scale data, this preprocessing requires too much time and waste memory.

**Fig 2:**
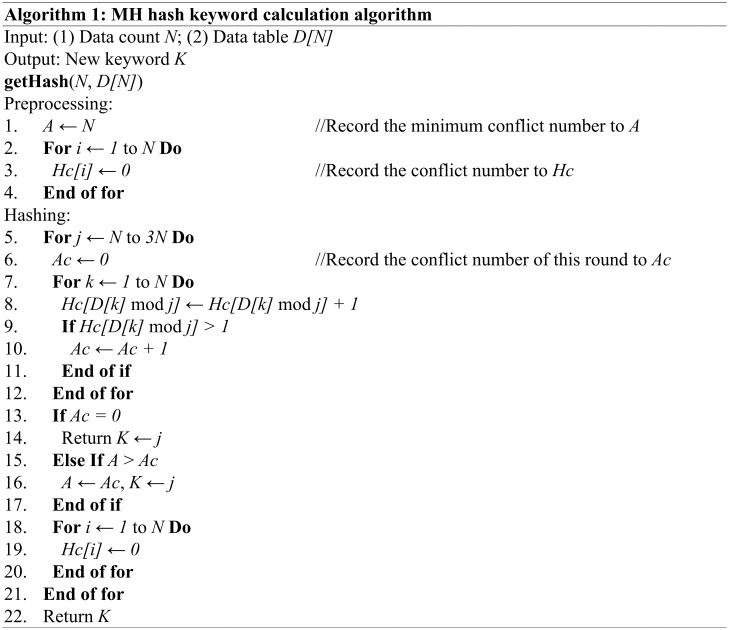
The simple hash method for exploring keywords.

To solve the above problem, we can perform some simple processing as follows. Set the initial keyword *K* to *N*, perform modulo *K* on the target data and record the average number *A* of conflict data. Then, set *K* to *K + 1* and repeat the above steps. When *A* is equal to 0, return *K*; otherwise, compare *A* with the average conflict number *Ac* of this round and update *A* and *K* if *Ac* is smaller than *A*; otherwise, continue to explore to *3N* (the exploration scope can be increased as needed). Then, save *K* to the previous node, hash the nodes in the current layer according to *K*, and save the conflict data with the same keyword into the conflict-binary table. This simple hash method for exploring the keywords is shown in Fig 2.

The algorithm above uses *N* temporary spaces, and its time complexity is *O(N*^*2*^*)*. This algorithm is simple but requires a long preprocessing time. Through observation and practice, we found that we can set a special keyword to reduce the preprocessing time in practical applications.

Therefore, a weight-based keyword algorithm was also explored to improve the matching speeds: This algorithm records the *i-th* node’s hit counts in the preceding time period as the weight *w(i)*, records the *i-th* node’s conflict counts in the *j-th* round as *cj(i)*, and can obtain the minimum conflict count *Acw(minimum)* and the corresponding keyword *K* according to Formula (1). (Here, *Acw(j)* represents the conflict weight in the *j-th* round (*N ≤ j ≤ 3N*)).

$$\left\{ \begin{array}{l} Acw(j)=\sum_{i=1}^{n}w(i)\cdot c_{j}(i)\\ Acw(least)=min\{Acw(N),Acw(N+1),\ldots ,Acw(3N)\}\\ K=\{j|Acw(j)=Acw(least)\} \end{array}\right..$$ (1)

This algorithm is reminiscent of the concept of a least-recently used (LRU) algorithm, which is widely used in operating systems—it dynamically adjusts the keyword by the matching status of the data flow over the preceding time period to achieve the best performance. However, in practice, the monitoring period setting needs to be tested and adjusted to suit various applications and the scale of the data.

#### Construction and destruction of hash-binary table chains based on digital sets

Based on the foundation set *Pd*, we establish the hash-binary tables layer by layer in the sequence *Ci1* to *Cisd(i)*. The nodes in the upper layer link to the next layer using pointers. Here, *Cix(1 ≤ x ≤ sd(i))* means the *x-th* digital node of rule *i*.

**Fig 3:**
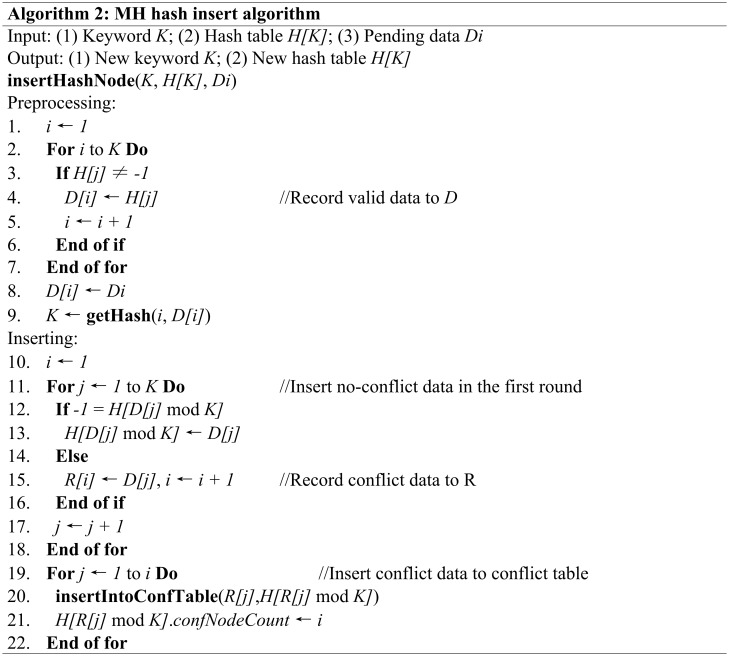
The algorithm for inserting data.

In the same hash table, data is stored using the same hash rules. When we need to insert data, we must first fetch all the original data. Next, we perform an exploration operation based on the algorithm shown in Fig 2. Then, insert the no-conflict data into the hash table and record the conflict data and, finally, insert the conflict data into the conflict-binary table sorted in ascending order and record the node count of the conflict-binary table in the upper layer nodes. This algorithm is shown in Fig 3. For existing nodes that equal *Cij*, we can skip the insert operation and operate on *Cij + 1* in the next layer.

This algorithm takes *N* temporary memory space, and its time complexity is *O(NM)*. The **insertIntoConfTable()** function inserts the data into the conflict-binary table in ascending order, and its time complexity is *O(M)* (where *M* is the number of conflict nodes).

To delete a rule *ri*, we need to remove the rule nodes in the last layer first to avoid the possibility of mistakenly deleting multiple rules in the same node. The rehash operation should be performed on the current layer when the nodes have sibling nodes and do not require backtracking; otherwise, the operation must to go back to delete the previous node in the upper layer and so on until the operation backtracks to the first layer.

**Fig 4:**
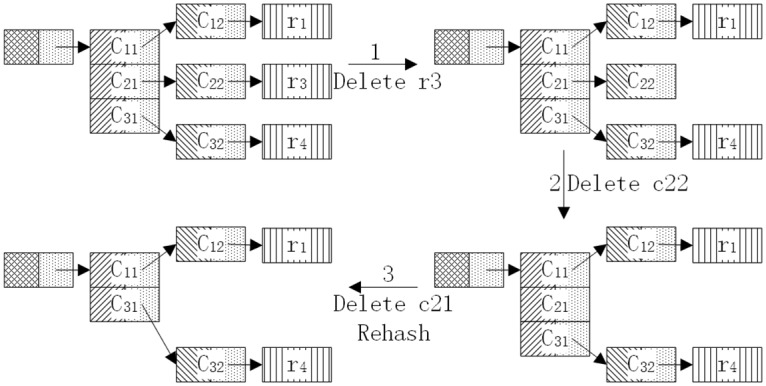
Delete rule *r3* from the hash-binary table chains.

Fig 4 shows the steps to delete a rule. When deleting the *3rd* rule from the chains, we need to delete the rule node *r3* first, then backtrack to the previous node *C22* in the upper layer. Because node *C22* has no sibling nodes in this example, we must backtrack to the parent layer and deletes node *C21*. That node has sibling nodes in the same layer; consequently, the rehash operation is required. The delete operation stops at this point. This entire removal process (except for the rehash operation) is similar to that used in HEM.

#### Incomplete node processing

When the PATH or HOST pattern string length is not an integral multiple of the machine word length, one or more characters that require less space than a machine word will remain (commonly called a remainder). We introduce a mask to solve this problem. We define the remainder set as *Re = {b1*, *b2*, *……*, *bn}* (*n ≤ lm*, where *lm* is the length of a machine word), which means the corresponding first *n* bits of the mask are 1, and the last *lm-n* bits are 0. Values of 1 in the mask indicates that the algorithm must address those bits, while values of 0 in the mask indicate that the algorithm does not need to be concerned with those positions.

The MH is a hash-based algorithm, the mask must be known before the hash algorithm; therefore, we add the mask field in the node structure to indicate the mask for the subsequent hash table. We introduce the minimum mask method to solve the problem of inefficiency and avoid too much mask use. The minimum mask method refers to recording the mask with the least possible number of 1's in the hash table and then performing the AND operation on all the data waiting for insertion. Then, we use those calculation results to insert the data into the hash table following the algorithm shown in Fig 3.

Generally, the mask problem described above exists only for the end node. The PATH and HOST nodes must set a mask tag and a subsequent mask status tag to identify whether the node is an end node. In the matching process, the target data must perform an AND operation on the end node and then perform the hash operation and compare it with the node. If the compare fails and the node counts are greater than 1, then the hash operation must be performed directly and then compared with the end node. In real-world situations, the probability of having to reperform this operation is very small.

We can add characters to the tail for the PATH node also. In an HTTP 1.1 data stream, the “*HTTP/1*.*1*” follows the PATH field, so we can extract a few characters in front of “*HTTP/1*.*1*” to supplement the remaining nodes if the length of the machine word is no more than 64 bits.

### Target data stream keyword positioning: HOST and PATH localization

The positions of the PATH and HOST strings in HTTP 1.1 streams are defined in the specification; therefore, we can locate the PATH and HOST quickly based on the specification and, thus, avoid the high costs of irrelevant information in the data flow and improve the performance of the matching process.

The set of main methods of HTTP 1.1 messages *Pm = {GET*, *HEAD*, *POST*, *PUT*, *DELETE*, *TRACE*, *CONNECT*, *OPTIONS}* is a mutex set in which we can usually determine the starting position of PATH by assessing only the first character (the first characters of the POST method and PUT method are both ‘*P*’, so the second character needs to be checked for those two cases).

As shown in Table 1, the PUT message’s PATH string starts at the *4th* character, while the HEAD message’s PATH string starts at the *5-th* character. Using the jump information shown in Table 1, we can quickly locate the beginning of the PATH field.

**Table 1:** Jump distance (starting position) for different PATH methods.

Method name	Jump distance (Starting position)
*GET/PUT*	4
*HEAD/POST*	5
*TRACE*	6
*DELETE*	7
*CONNECT/OPTIONS*	8

HOST field positioning using the “Host:” string in target HTTP 1.1 streams starts from the *2nd* character after the colon (“:”). We can use the classic single pattern matching algorithms or traverse backward directly; either method has only a small effect on performance because the data flow will be mismatched at the PATH field in most cases.

### Fast matching for pattern strings

**Fig 5:**
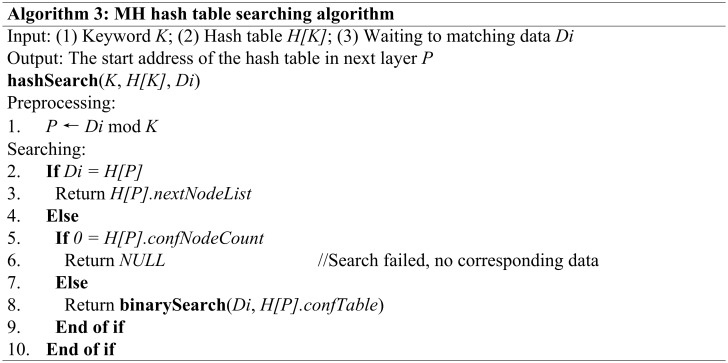
The algorithm to search the corresponding hash table of the target data.

After this pretreatment of the pattern strings and the positioning of the PATH and HOST in the data streams, we can retrieve the matching rule from the hash-binary chains efficiently. In the search process, the matching of the HOST field occurs after the matching of the PATH field and the entire process is completed using the multiple hash and binary searching processes. It is important to note that we must compare the node’s value with the target data if an address is matched using a single hash search, and we must perform a binary search if the node has a conflict table and the target data are not equal to the node’s value. This search algorithm is shown in Fig 5.

This algorithm’s space complexity is *O(1)*, and its time complexity is *O(logM)* when the number of nodes in the conflict table is *M*, or *O(1)* when no conflict occurs. The **binarySearch()** function searches for the target node in the conflict table using the binary search method.

**Fig 6:**
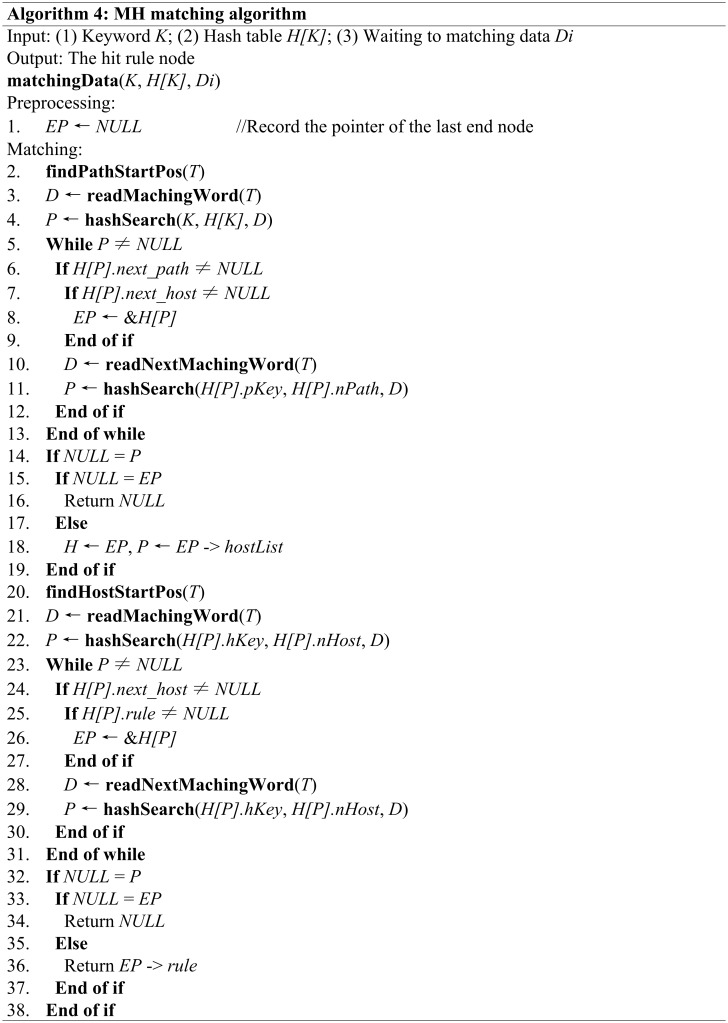
The complete matching algorithm of the MH.

MH is a depth matching algorithm; namely, the algorithm performs forward matching when the data hits an end node that has subsequent nodes, and it returns the Rule ID in the last matched HOST end node. During the matching process, the previous end node must be recorded because when matching fails, the algorithm must be able to backtrack to the last end node and continue searching (or return null directly when no backtracking is needed) to improve the processing speed. The complete matching algorithm is shown in Fig 6.

The space complexity of this algorithm is *O(1)*, and its time complexity is *O(N)*. The **findPathStartPos(*T*)** and **findHostStartPos(*T*)** functions find the start position of the PATH and HOST fields, respectively, in the target stream *T*, and the **readMachingWord(*T*)** and **readNextMachingWord(*T*)** functions retrieve a machine-word-length string from the target stream *T*.

#### PATH data matching

After locating the start of the PATH position in the target data stream *T*, the algorithm converts a machine-word-length data string at the start of the PATH to an unsigned integer and use that to perform a search in the first layer. When the data matches, the algorithm continues to convert and search for the next machine-word-length string until the last PATH end node is found. Then, it retrieves the corresponding HOST hash table and begins the steps to match the HOST.

#### HOST data matching

After obtaining the HOST table, the algorithm locates the HOST position in *T* and then performs a hash and binary search process similar to those performed for PATH data matching. It returns the Rule ID when it finds the last HOST end node. The end node search involves performing the mask operation, meaning that an AND operation must be performed between the machine-word-length string of the HOST in the target stream *T* and the end node’s value. Then, the hash search is performed using the result.

#### Backtracking for the common prefix end node problem

During the matching process, if the node is an end node of some rules but is also a prefix node of another rule, the common end node mismatch problem occurs because we cannot be sure which rule will be matched. As shown in Table 2, when the data stream “www.test.com/00003” arrives, Rule 1 will be matched first, but there are more precise rules (Rules 2–4); consequently the matching process needs to continue. When matching to character “3”, Rule 4 matches, but Rule 4 contains the subsequent character “1”; therefore, matching fails, and the algorithm needs to backtrack to the last node of Rule 1 (the common end node). At that point, the algorithm completes, and Rule 1 was matched.

**Table 2:** A group of rules requiring backtracking.

Rule ID	Rules
1	www.test.com/0000
2	www.test.com/00001
3	www.test.com/00002
4	www.test.com/000031
5	www.test/00001

To ensure correct matching, the algorithm needs to record the pointer to the last common end node and the corresponding position in the stream. Then, during the matching process, the algorithm must backtrack to those recorded nodes to execute the next steps.

Because the last common end node has been recorded, there is no need to perform hash matching again when a node mismatch occurs; therefore, the overall matching efficiency is not significantly decreased. In practice, the common end node mismatch problem is uncommon and requires only a small amount of time overhead. In addition, we can add a space after the PATH node to reduce the need for backtracking.

#### More compact data structures

In practical applications, it was found that the URL set obviously converges according to the layer—URLs with the same prefix that occur less often should be placed later and ordered based on which URLs have fewer corresponding subsequent nodes. The count of the nodes in the last layer is 1. Therefore, if we build the hash table so that these occur before the nodes with several layers and then build the binary table for the subsequent nodes, we can get a good effect and greatly reduce the pretreatment time.

#### MH automaton model

**Fig 7:**
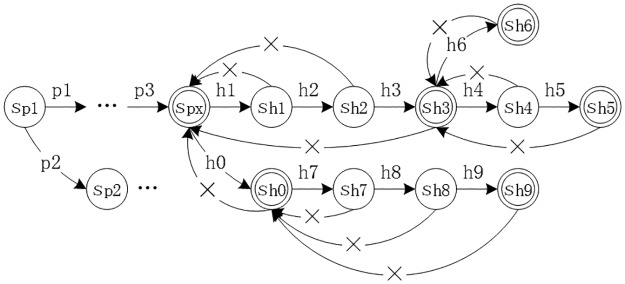
MH automaton model.

In general, MH is a deterministic finite automaton algorithm. The automaton model is shown in Fig 7, each node state (shown by circles) can jump to the subsequent state, and an end node (shown by double circles) corresponds to an end state. The “×” symbol indicates that if the state jump fails, the algorithm backtracks to the last matching end state in the sequence (only one backtracking event occurs for each mismatching event).

### Algorithm complexity

The MH algorithm improves the matching speed using data pretreatment. Here, we focus on the time complexity of the matching process. We assume that the number of rules is *M*, the machine word length is *lm*, the *i-th* rule’s length is *lr(i)*, the rule number added before the *i-th* rule is *Mi*, the number of corresponding machine words in the longest rule is *m*, and the average number of machine words is $ma=\frac{1}{M}\sum_{i=1}^{M}Integer(\frac{lr(i)}{lm})$.

#### Preprocessing time complexity

To simplify the preprocessing, we add the rules item by item. In the processing involved in adding a rule, considering the need for multiple hashes and binary searches and inserts, the time complexity tends to *O(1)* for one hash insert and *O(N)* for one binary insert; therefore, the average complexity of one insert is approximately *O(maM)*, and the time complexity for adding *M* rules is approximately $O(M\sum_{i=1}^{M}(\frac{lr(i)}{lm}))\leq O(mM^{2})$.

#### Matching time complexity

In a single matching process, assume that the total number of data values is *N*. If we adopt the pure binary-matching method, the time complexity is *logN*. If, instead, we adopt the hash-binary method which with load factor t and table length M, the algorithm’s time complexity can be reduced to *log(N/Mt)*. When the hash table length M tends toward N, and the load factor t tends toward 1, the value of *Mt* tends toward N and the actual match number tends toward 1, which is the greatest advantage of the hash method.

However, the matching processes for PATH and HOST includes several hash and binary searching sub-procedures. Because the single algorithm’s time complexity tends toward *O(1)*, the algorithm’s time complexity tends toward *O(m)* when the longest rule string length is *m×lm*.

In HTTP 1.1 messages, the PATH and HOST fields account for only a fraction of the entire message; consequently, localization of the PATH and HOST, for which the time complexity is *O(n)*, generally occupies the most time. However, the matching probability is very low in most applications, the algorithm is likely to mismatch the most in the PATH field; consequently, the complete time complexity tends toward *O(m)*. Compared with the other classic multi-pattern algorithms such as AC, because the MH algorithm uses a machine-word matching method, its speed will obviously increase. Compared with binary search algorithms such as HEM, MH only tends to need a single hash search, accruing additional advantages in time complexity.

#### Space complexity

The algorithm must establish the hash-binary table chains for the rules during the preprocessing step. The load factor of the hash tables is between 0.33 and 0.5, the space complexity for processing is *O(N)*, and it only a machine-word-length temporary space is required during the matching process to record common end nodes. Therefore, the algorithm’s space complexity tends toward *O(mM)*. In reality, because of the existence of common nodes among the rules, the space costs are less than *3mM* machine words.

Compared with the HEM algorithm, which is based on pure binary tables, the MH algorithm uses the hash method to store the nodes, which involves little wasted space and overall, is a strategy to trade space for time. In our algorithm, limiting the maximum exploration range to *3N* reduce wasted space and can still meet the performance demands in most cases. If there is a need for faster matching and enough memory is available, the maximum exploration range can be extended.

## Experimental evaluations

We performed multiple matching speed and memory consumption tests to compare 9 algorithms (MH, HEM, AC, WM, SBOM, M-BNDM, EPSM, EXHAUST and SUMSMF, denoted as COMMON) in a common development environment. And we performed matching speed tests to compare 6 algorithms (BXS, BP2WW, KBNDM, SSECP, FSBNDM and BSDM, denoted as SMART) based on the string matching research tool (Smart). Experiments showed that the MH algorithm achieved a good performance under a wide variety of scales and rule sets.

### Data and environment

#### Experimental data

We grabbed 3.76 GB of HTTP data packets on the network. These packets contain a total of 10,441 different URLs, from which we randomly selected 10,000 URLs to form the main rules. Then, we randomly generated 100,000 URLs as alternative rules. Based on these data, we established 3 groups of sets: Set A contained 5,000 main URLs (whose hit rate was 100%), set B contained 10,000 main URLs (whose hit rate was 100%), and set C contained 20,000 URLs (10,000 main URLs and 10,000 alternative URLs whose hit rate was 50%).

To obtain a more comprehensive performance evaluation of MH and make a thorough comparison with HEM, SBOM, M-BNDM and SUMSMF, we established 10 group sets (Sets 1–10), containing 10,000–100,000 URLs, respectively. Each set contained 10,000 main URLs (whose hit rate ranges between 100% and 10%). For the various algorithms provided by Smart, we also established 11 other group sets (U1, U10, U50, U100, U500, U1000, U2000, U4000, U6000, U8000 and U10000), which contained 1, 10, 50, 100, 500, 1,000, 2,000, 4,000, 6,000, 8,000 and 10,000 main URLs (hit rate 100%), respectively.

#### Experiment environment

For the COMMON algorithms, we adopted a common development environment for the test machine, consisting of an Intel Core i7-4770 (8 cores, 3.40Ghz) CPU and 12.0 GB memory running the Windows 7 Ultimate operating system. All the COMMON algorithms were implemented in C and compiled using Eclipse and MigGW, and all were executed in single-threaded mode. For the SMART algorithms, we adopted a test machine which consisted of an Intel Core i7-6700K (8 cores, 4.00Ghz) CPU and 16.0 GB memory running the Ubuntu 14.04 desktop operating system. All the SMART algorithms were implemented in C and compiled with GCC.

Each group of experiments was repeated more than 10 times; their average results were taken as the compared data. The matching time ignored all the pretreatment time for each data packet (including judging the protocol of the target data stream, unpacking the data, port and IP address analysis, and so forth)—for the comparisons, we considered only the pure matching time.

### Comparison of the experimental results

#### Matching speeds (COMMON)

The algorithms speeds are shown in Table 3. For all the rule sets, MH consumed the least amount of time, and HEM achieved second place. As the number of rules increased, the difference between MH and HEM decreased, but their performance advantages become greater compared to the other algorithms. In our experiments, the performance of the AC algorithm obviously degenerated as the number of rules increased.

**Table 3:** Matching speed of each algorithm in 3 rule sets (MB/s).

Algorithms	Set A	Set B	Set C
HEM	3,565.56873	2,927.05478	2,748.60565
SBOM	82.15844	38.17939	38.20286
WM	38.26061	18.63397	15.29269
AC	0.03370	0.00807	0.00569
M-BNDM	246.05880	125.55303	64.74252
EPSM	4.29586	2.06469	1.07239
EXHAUST	68.42028	33.62729	33.59795
SUMSMF	125.27794	124.54173	115.14880
MH	**4,090.81034**	**3,313.57698**	**3,333.14238**

As Table 3 shows, M-BNDM and SUMSMF achieve the next closest match speeds to MH and HEM. When there are fewer rules in the set (Set A), M-BNDM is approximately 17 times slower than MH, and as the number of rules increased, its performance degraded significantly. SUMSMF is approximately 30 times slower than MH on Set A. As the number of rules increased and the hit rate decreased, SBOM, WM, EXHAUST and SUMSMF gradually converged, as did MH. Among them, SBOM is 80 times slower than MH, EXHAUST is more than 60 times slower than MH.

#### Memory consumption (COMMON)

As shown in Table 4, the memory consumed by HEM, SBOM, WM, AC, EPSM and EXHAUST basically grew linearly. The M-BNDM and SUMSMF algorithms initially consume more memory, but require only a small memory increase in follow-up, depending on the efficiency with which they manage rule nodes. Among all the algorithms, WM consumed the smallest amount of memory, HEM was second, MH was third, and EXHAUST was fourth. However, there was no huge gap between these algorithms. AC not only consumed the largest amount of memory but also, as the number of rules increased, its memory consumption became increasingly unaffordable.

**Table 4:** Memory consumption of each algorithm in 3 rule sets (MB).

Algorithms	Set A	Set B	Set C
HEM	0.86328	1.84766	6.31250
SBOM	84.36719	169.05078	338.64844
WM	**0.70313**	**1.40625**	**2.90234**
AC	188.05469	375.35938	851.86719
M-BNDM	92.60938	93.61068	95.90625
EPSM	20.30078	40.54688	80.91406
EXHAUST	4.23047	5.57943	8.31901
SUMSMF	33.29167	33.84896	34.98828
MH	1.37891	2.74601	7.49219

#### Comparison of MH, HEM, SBOM, M-BNDM and SUMSMF

For a more comprehensive reflection of the real differences and performance gaps among MH, HEM, SBOM, M-BNDM and SUMSMF, we conducted a series of tests using Sets 1 to 10 as described above.

**Fig 8:**
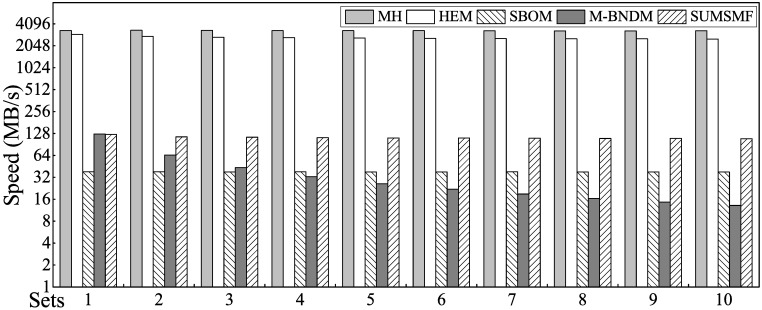
Matching speed comparison of MH, HEM, SBOM, M-BNDM and SUMSMF.

**Fig 9:**
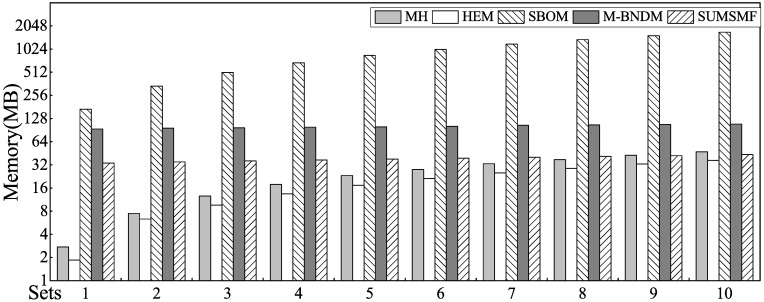
Memory consumption of MH, HEM, SBOM, M-BNDM and SUMSMF.

The matching speeds and memory consumption of the MH, HEM, SBOM, M-BNDM and SUMSMF algorithms are shown in Figs 8 and 9, respectively. The digitally-based algorithms (MH and HEM) exhibited huge performance advantages in matching speeds compared with the other algorithms. In terms of memory footprint, when the number of rules is less than 60,000, MH and HEM algorithms have obvious advantages; the HEM algorithm performs best, and SUMSMF requires less memory than MH when the number of rules exceeds 80,000. In these algorithms, the memory consumption of the M-BNDM and SUMSMF algorithms is relatively stable, due to their excellent memory management strategies.

**Fig 10:**
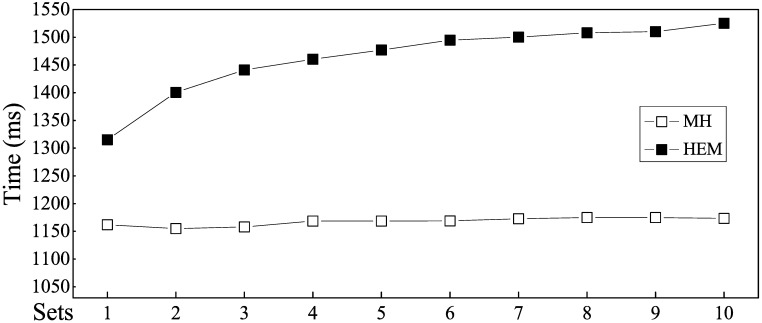
Matching speed comparison of MH and HEM.

Fig 10 reflects the differences only between MH and HEM. As the number of rules increased, the matching speed of HEM shows a logarithmic growth. This characteristic reveals an important advantage of HEM and accords with actual binary search characteristics. In contrast, the advantage of MH lies is its stability; namely, no matter how large the number of rule becomes, the algorithm shows a stable matching performance.

**Fig 11:**
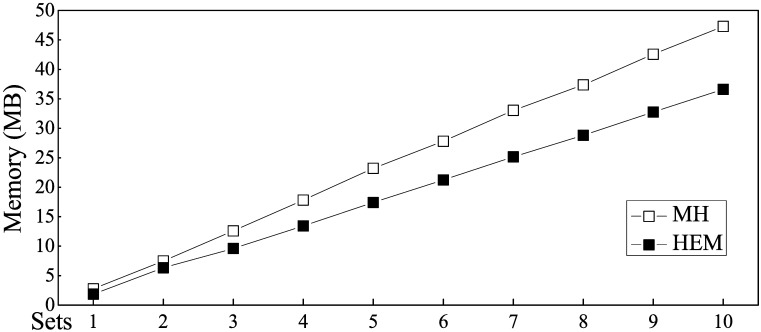
Memory consumption comparison of MH and HEM.

The memory consumption of HEM and MH is shown in Fig 11. The memory required by both MH and HEM grew linearly: memory consumption increased along with the number of rules. The main memory consumed included the temporary cache for the pre-read pattern string and the converted digital nodes. As shown by the line chart, MH required more memory than HEM because of its hash-based storage methods, while HEM uses a sequential storage mode with no wasted space.

The experiments showed that MH and HEM are much faster than SBOM, M-BNDM and SUMSMF in all cases and that both MH and HEM each have specific advantages. When less memory is available, HEM is the most appropriate algorithm; otherwise, MH is more advantageous in most cases.

According to the comparison data given in [28], MH is also approximately 40 times faster than SOGOPT, requires less memory space, and has significant performance advantages in matching URL fields.

#### Matching speeds (SMART)

Based on the rule sets U1, U10, U50, U100, U500, U1000, U2000, U4000, U6000, U8000 and U10000, this paper compared the algorithms of BSDM, SSECP, FSBNDM, BXS, KSA and KBNDM provided by the Smart tool. As the results in Table 5 show, the gap between these algorithms and MH is small when there are few rules. Especially in the case where there is only a single rule, FSBNDM’s matching speed is slightly faster than MH.

**Table 5:** Matching speed of each algorithm in the SMART group (MB/s).

Sets	BXS	BP2WW	KBNDM	SSECP	FSBNDM	BSDM	MH
U1	13,565.89	14,506.41	12,038.77	14,354.96	**20,522.39**	9,836.48	18,078.51
U10	1,424.66	1,401.63	1,079.58	1,247.57	2,176.12	1,050.31	**17,272.32**
U50	269.96	275.37	243.02	266.25	418.23	216.07	**16,328.78**
U100	133.91	136.36	124.74	124.11	207.48	107.15	**15,847.53**
U500	27.83	26.74	25.41	30.39	43.25	22.22	**13,220.25**
U1000	13.98	13.82	12.51	15.35	21.67	11.16	**11,764.35**
U2000	7.07	7.12	6.61	8.60	11.13	5.68	**10,206.79**
U4000	3.61	3.67	3.30	3.81	5.76	2.87	**8,430.04**
U6000	2.33	2.41	2.19	1.78	3.72	1.91	**7,678.75**
U8000	1.75	1.80	1.61	1.65	2.78	1.41	**7,019.19**
U10000	1.41	1.41	1.27	1.34	2.20	1.11	**6,522.06**

However, when the number of rules is large, the matching speeds of each algorithm in the SMART group is low, and as the number of rules increases, their performance curves decrease linearly. This result occurs because Smart uses serial processing and is not optimized for multiple rules.

Because these algorithms use the unified memory management of Smart, we only compare the matching speeds of these algorithms.

## Conclusions

MH is the second high-efficiency algorithm the authors have proposed; HEM was the first. This algorithm transforms the symbol-space matching problem into a digital-space numerical-size comparison problem, and it effectively combines both the basic features of the HTTP protocol and URL patterns. In addition, MH proposes using a hash method combined with a binary search method to match the target data, an approach that improves the matching speed while requiring less memory overhead.

### Advantages and limitations

The experiments showed that MH is more efficient than HEM, and it has a faster matching speed and consumes less memory than the classical algorithms in the HTTP stream-matching field. Meanwhile, the MH algorithm has relatively stable memory consumption and no obvious degradation; these are also desirable features to which the classical algorithm cannot compare. Based on the comprehensive comparison with HEM, MH uses a time-for-space trade-off strategy and, therefore, is more extensively applicable in today’s hardware conditions.

However, MH is solely a URL-matching algorithm for HTTP stream fields, it is intended for use in a variety of network device fields: network software, robot communications, web server load balancing, big data collection and processing, and so on, but it is (temporarily) unable to be used for general string matching purposes. In addition, MH requires a longer preprocessing time and more memory than HEM.

### Future research

In recent years, as big data, cloud computing and even cloud robotics have developed, the requirements for faster matching speeds have been increased. Such fields each have their own applicable protocols, and MH is very suitable for fields that follow specific protocols and whose data streams conform to specific data formats. In these protocols, there is no need to spend much time to find the starting position of a field; instead, data matching can start from a known fixed position. Therefore, adopting MH in these fields will improve the matching performance.

Next, we will further analyze URL features and those of various protocol types and combine these with the probability weighted method [32], to explore new hash algorithms that exhibit lower conflict rates, achieve faster matching speeds and consume less memory. Furthermore, we plan to adapt MH for use in communication and for nodes discovery in cloud robotics and will also try to use the idea of string conversion to machine words for generic string processing to improve the speed and practicability of classic algorithms.

## Supporting information

S1 Data Initial data of this paper. (XLSX)
